# Descriptive Report of a Pharmacist-Directed Preconception Care Outreach Program in a Rural Maternity Care Desert

**DOI:** 10.3390/pharmacy11060176

**Published:** 2023-11-10

**Authors:** Natalie DiPietro Mager

**Affiliations:** Raabe College of Pharmacy, Ohio Northern University, 525 S. Main St., Ada, OH 45810, USA; n-dipietro@onu.edu; Tel.: +1-419-772-3971

**Keywords:** pharmacists, students, pharmacy, preconception care, women’s health, rural health, mobile health units

## Abstract

Preconception care is the prevention and management of biomedical, behavioral, and social risk factors to improve pregnancy outcomes and overall health for reproductive-age patients. A community-based pharmacist-directed preconception care outreach program was developed for women ages 18–45 years living in a rural maternity care desert to help them identify potential health risks and provide them with the needed education, counseling, or referrals to address these risks. Supervised student pharmacists, pharmacy practice residents, and pharmacy faculty from a local University collaborated to provide this program at four community events in conjunction with a mobile health clinic. A summative evaluation was performed after the events concluded, modeled after the RE-AIM framework. One hundred and forty-one women were served by the outreach program. Nearly 98% reported at least one preconception health risk, and 45% reported a barrier preventing them from being able to have an appointment with a physician in the last year. The outreach program was feasible to implement and can be adapted to different settings. Pharmacist-directed outreach programs in rural communities may benefit patients who are not receiving or do not have access to such care in traditional healthcare settings.

## 1. Introduction

Preconception health refers to the health status of reproductive-age people before pregnancy. Interconception health, or health between pregnancies, is a subset of preconception health. It is important that all people of childbearing age have good preconception health regardless of pregnancy intention. Identifying and managing biomedical, behavioral, and social risk factors in the preconception period is known as preconception care, and is a key strategy to improve health and pregnancy outcomes [1,2,3]. Preconception care is recommended to be incorporated into primary care for all patients of childbearing potential in the United States, as nearly half of all pregnancies are unintended [4].

Given there are a wide range of considerations to ensure the provision of comprehensive preconception care, the United States Centers for Disease Control and Prevention (CDC) released a core list of topic areas to be routinely assessed and addressed with reproductive-age women: health conditions (i.e., diabetes, HIV/AIDS, hypothyroidism, maternal phenylketonuria, obesity, sexually transmitted infections), teratogenic medication use, vaccinations (i.e., rubella, hepatitis B), and health behaviors (i.e., alcohol misuse, folic acid use, smoking) [1]. Pharmacists and student pharmacists in the United States currently receive education and training to be able to address each of these topics with patients alone or as members of interdisciplinary teams, either through direct provision of care, education, counseling, or through referral to other healthcare providers [5,6,7,8,9,10].

One of the goals of the CDC Preconception Care National Health Plan is to ensure that all women receive “screening, health promotion, and interventions that will enable them to achieve high levels of wellness, minimize risks, and enter any pregnancy they may have in optimal health” [11]. Currently, many women in the United States do not receive recommended preconception health services; studies in the United States have shown that reproductive-age women with chronic conditions often do not receive appropriate preconception health counseling or disease state management from their healthcare providers [12,13,14].

Good preconception health is important for all women, even those who do not desire children or have finished childbearing since the dual purpose of preconception care is to improve women’s personal health as well as to minimize the potential risk of adverse outcomes should pregnancy occur [1,2,3]. For example, given its negative effects during pregnancy, tobacco use is considered a health risk to address through preconception care but it is also important to discuss with all women as it can have severe consequences for their overall long-term health [15]. Another example is daily multivitamin use; while the folic acid component of the multivitamin is important to prevent birth defects such as neural tube defects, other components of the vitamin (e.g., calcium) may help to prevent long-term health concerns for women (e.g., osteoporosis) [16].

Pharmacists and student pharmacists can play key roles in delivering preconception care, and there are documented examples of pharmacist provision of preconception care services and risk assessment in community pharmacies [5,6,7,8,9,10,17,18,19,20,21]; however, not all community members may have access to healthcare facilities. Of special concern is providing preconception care to individuals living in underserved areas also known in the United States as health professional shortage areas or maternity care deserts, where there is a dearth of primary care or obstetric providers, respectively [22,23]. In such cases, outreach events can be an important way to provide education and raise awareness on health issues as well as to provide care for community members who do not have access to, or who do not routinely access, the health care system [24]. Residents of rural counties in the United States where there is low population density are more than twice as likely as residents of urban counties with high population density to face challenges related to healthcare accessibility, affordability, and quality [25,26,27]; therefore, individuals living in rural, underserved areas may especially benefit from outreach events taking place in their local communities. The purpose of this project was to examine the feasibility and potential impact of a pharmacist-directed preconception care outreach program in a rural maternity care desert.

## 2. Project Description

### 2.1. Setting

Hardin County is a rural county in northwest Ohio with a population of about 30,400 people. The county seat is the largest town in the county, with a population of approximately 7800. The next-largest settlement is a village of about 5200 people where Ohio Northern University is located [28]. Hardin County is classified as a Health Professional Shortage Area by the Health Resources and Services Administration and a maternity care desert by the March of Dimes [22,23]. Hardin County’s overall Maternal Vulnerability Index has been rated as 81.9, or “very high”, indicating that compared to the average woman in the state of Ohio, women in Hardin County are more vulnerable to adverse maternal health outcomes due to county-level conditions [25].

### 2.2. Outreach Program Design

Since 2015, pharmacists and student pharmacists have sought to fill gaps in care for all residents of Hardin County through the use of a mobile health clinic. The mobile health clinic is operated by faculty, residents, and students from Ohio Northern University’s pharmacy program in Hardin County working in conjunction with medical resident physicians from Mercy Health St. Rita’s Hospital in Allen County. The mobile health clinic provides a number of no- or low-cost primary care services including point-of-care screening (e.g., blood pressure, cholesterol, glucose, hemoglobin A1C, hepatitis C); immunizations; tobacco cessation; chronic disease management (e.g., diabetes, hypertension, hyperlipidemia); and health education. The mobile clinic travels to communities within Hardin County and surrounding counties on various days of the week [29,30,31].

However, uptake of services provided by the mobile health clinic among reproductive-age women had been slow; therefore, this program aimed to raise awareness among women in Hardin County about the availability of the mobile health clinic while providing them personalized education and referrals as appropriate to improve their health and the health of a future pregnancy. Finally, another intention of the program was to give student pharmacists opportunities to perform patient interviews and counseling and to provide them insight into geographic health disparities.

The outreach program was developed in consultation with the local health department and its Healthy Lifestyles Coalition, which is a partnership of local community organizations focused on planning, implementing, and promoting prevention initiatives to improve the health of the community [32]. The program followed the model proposed for preconception health services: (1) risk identification; (2) education; and (3) intervention [33].

The outreach program sought to bridge preconception health needs identified at national, state, and local levels. It was decided to use the recommendations from the CDC as the focus of the program; therefore, health conditions that should be well-controlled before and during pregnancy, receipt of recommended vaccines for reproductive-age women, folic acid use, alcohol consumption, tobacco use, and physical activity were addressed [1]. At the state level, Ohio’s State Health Improvement Plan lists among its priorities to improve health for Ohio residents with a focus on maternal and infant health, health behaviors such as tobacco use and lack of physical activity, and local access to healthcare providers. [34]. The most recent Hardin County Community Health Assessment found that 11% of adults were without healthcare coverage and 19% described their health as fair or poor. It further found that only about 66% of women aged 21–65 years had received a Pap test in the past three years, a proxy for routine preventive health service use, as compared to 82% for Ohio women overall. Addressing chronic disease and access to care (i.e., primary care providers, mental health providers, obstetricians/gynecologists) were listed as two priorities for the county [35]; therefore, barriers preventing an appointment with a physician were also captured.

Non-pregnant women between the ages of 18 and 45 years who were permanent residents of Hardin County were the intended participants of the program, regardless of pregnancy intent. Since women who are not actively planning a pregnancy may not be as receptive to preconception care as they may think it does not apply to them, the previous literature recommended that these services be referred to as promoting “healthy lifestyles” or “women’s health” [36]. Therefore, this project was framed as a “women’s health” outreach program when it was implemented in the community.

To ensure a standard experience for each participant, prior to delivering the program in the community the volunteers (i.e., pharmacy faculty and PGY-1 pharmacy residents who were serving as preceptors, and student pharmacists) attended overview sessions where they learned about the flow and process for the events. The questions on the form were also reviewed to highlight answers that would warrant education or referral and local community assets that could be helpful to patients.

The program was implemented by pharmacists, pharmacy practice residents, and supervised student pharmacists at four community events such as festivals and fairs in the county. The mobile clinic van was parked prominently among other vendors and booths. The volunteers stood outside of the van, greeting and approaching women who appeared to be non-pregnant and in the desired age range to assess their interest in participating in the program.

Those who chose to participate were asked to complete a brief, one-page questionnaire (Appendix A) assessing demographic information, health conditions, and health behaviors based on the preconception care recommendations from the CDC to identify patient-specific risks [1]. Questions on the form were adapted from the Behavioral Risk Factor Surveillance System (BRFSS) and Pregnancy Risk Assessment Monitoring System (PRAMS) surveys utilized by the CDC as well as the Ohio Pregnancy Assessment Survey (OPAS) utilized by the Ohio Department of Health to collect data on health conditions, behaviors, and barriers to care [37,38,39], and was a shortened version of a longer form that was used in a cross-sectional study to quantify health needs for reproductive-age women living in this area [40].

Of the measures collected on the questionnaire, seven are considered tier 1 preconception health indicators by the National Preconception Health and Health Care Initiative’s Surveillance and Research work group. Tier 1 preconception health indicators are considered the most important measures for states to collect and track in order to improve health for reproductive-age women [41]. The seven tier 1 preconception health indicators addressed with this outreach program were diabetes, heavy alcohol consumption, hypertension, folic acid intake, normal weight, physical activity, and tobacco use.

After each participant completed the questionnaire, the pharmacist or supervised student pharmacist reviewed the information with her. Specifically, each participant was provided verbal counseling to address any identified health needs and a written form summarizing this information (Appendix B). Referrals were also made, as appropriate, based on individual needs and could include the local health department, University mobile health clinic and/or community pharmacy, or mental health and recovery services. Any participant who marked on the questionnaire that she would like additional information on how to have a healthy baby received an additional handout and more detailed counseling (Appendix C).

After the four outreach events were implemented, a summative evaluation was performed modeled after the RE-AIM framework (reach [R], effectiveness [E], adoption [A], implementation [I], and maintenance [M]) to determine whether the program should be offered again [42,43,44]. Descriptive statistics were utilized to characterize the demographics, health conditions, health behaviors, and healthcare barriers reported by recipients. Approval to disseminate the results from this outreach program was obtained from the Ohio Northern University Institutional Review Board. All participants provided verbal informed consent for their data to be shared in an aggregate format.

## 3. Findings

### 3.1. Reach

“Reach” indicates the absolute number of individuals who participate in the program and the representativeness of those participants for the community the program is working to serve [42,43,44]. One hundred and forty-one women were served through the outreach programs. Table 1 shows the demographic profile of the participants. All women were full-time residents of Hardin County, and their mean age was 31.5 years (standard deviation ±7.2 years). The racial and ethnic distribution was similar to the overall racial and ethnic profile for the county based on Census data where 93.6% of the population is white, non-Hispanic, and 2% are two or more races [28].

### 3.2. Effectiveness

“Effectiveness” refers to the impact of the intervention, which can be sometimes difficult to assess in a community-based program [42,43,44]. A limitation of outreach programs such as this is the inability to fully track the impact of the program or long-term outcomes for participants over time, limiting the ability to fully evaluate the program’s effectiveness. However, the baseline measures collected from participants in the program are reported here, as they show the current depth and breadth of unmet preconception health-related needs, the topic areas for which participants received education and/or referrals, and potential areas to assess for effectiveness moving forward.

Program participants reported they had been diagnosed with health conditions including overweight/obesity (79.4%), thyroid problems (10.6%), hypertension (9.2%), seizures/epilepsy (3.5%), or type 1 or 2 diabetes (2.1%). Each of these chronic medical conditions can be managed by a pharmacist and should be well-controlled to optimize women’s health and maternal–fetal outcomes. Women also reported modifiable health behaviors that pharmacists can impact including lack of sufficient physical activity (75.2%), not taking folic acid or a multivitamin every day (66.7%), not receiving a flu vaccine in the past year (54.6%), and using tobacco products (20.6%). Based on the answers women provided on the questionnaire, they received education and counseling as well as referrals to community resources as needed. For example, women who indicated that they smoked were strongly encouraged to utilize the free tobacco cessation services provided by the mobile health clinic.

The total number of tier 1 preconception risks reported by each participant was counted (Table 2). Only 2.1% of women reported no tier 1 preconception health risks. Nearly 10% reported one health risk, 32.6% reported two health risks, 46.1% reported three health risks, and 9.2% reported four or more health risks. The majority of women reported at least two health risks, which emphasizes the importance of comprehensive primary care for reproductive-age women.

Women who participated in the outreach events were also asked about barriers preventing them from receiving a physician appointment in the last year. About 45% of women reported at least one barrier (Table 3). Some of the barriers reported can be alleviated using outreach programs such as this one.

Finally, 7.1% of participants reported wanting more information on family planning and received education and referral. Only 9% of participants reported wanting information on how to have a healthy baby and received additional counseling and education. The small percentage requesting additional information about healthy pregnancies seems to indicate that an outreach program exclusively marketed as focused on preconception health would not draw much interest.

While this outreach program provided education, counseling, and referrals as needed regarding preconception health to 141 women, there are limitations. One limitation is that data were self-reported, and women were asked on the questionnaire whether they had been diagnosed with certain health conditions. Since almost half reported a barrier to a physician appointment in the last year, it is possible that women may have health problems that were not yet diagnosed. Additionally, the prevalence of preconception health measures and barriers to care collected during these outreach events may not represent the true prevalence of these conditions among all reproductive-age women in Hardin County; however, the data gathered do show substantial need among at least some community members that pharmacists and student pharmacists can help to address. Over time, if participants in this outreach program begin to regularly use the services of the University mobile health clinic or community pharmacy, there will be the potential to track health outcomes longitudinally. Other opportunities to assess effectiveness in the future include obtaining participant and stakeholder opinions of effectiveness through focus groups, interviews, or surveys [42,43,44].

### 3.3. Adoption

“Adoption” signifies the number of settings and providers who participated in the project and whether these are representative of what the community uses [42,43,44]. The outreach program was implemented at four community events in Hardin County (i.e., county fair, festivals) in conjunction with the services of the mobile health clinic. Five pharmacists, two PGY-1 pharmacy practice residents, and over 25 student pharmacists worked together to deliver the outreach events. Over the last decade especially, the College of Pharmacy has intentionally increased its outreach programs in the community such that it is common for them to be at community events providing education and counseling on a wide variety of topics. While this was the first time a program specifically on women’s health or preconception health was implemented, community members are familiar with similar programs and often participate.

### 3.4. Implementation

“Implementation” examines whether the program was consistently delivered or whether adaptions were needed [42,43,44]. It was found that no changes to the program were needed, as it was able to be delivered as intended. The pharmacists and student pharmacists who volunteered had the skills and training to provide education, counseling, or services to women to address the health risks that were identified.

### 3.5. Maintenance

“Maintenance” is the extent to which the program becomes a part of the usual practices of the organization, as well as individuals, to sustain its effects [42,43,44]. This program is one that can continue to be offered by the University to community members. While individual maintenance is hard to quantify at this point, it may be able to be tracked over time through effectiveness measures as detailed above.

## 4. Implications

This project report describes an outreach program focused on preconception health in a rural, medically underserved community designated as a maternity care desert. It is estimated that up to 6.9 million women in the United States currently live in areas where there is low or no access to obstetric services [23]. Pharmacist-directed programs such as this one may help to address unmet needs in these populations. While previous reports of pharmacist-provided preconception health interventions have occurred mainly in community pharmacies and have tended to focus on singular aspects of preconception health (e.g., smoking cessation, iron or folic acid supplementation, vaccination status, use of teratogenic medications) [17,19], screening for health risks [20,21] or providing health education [45,46], this is the first published paper to describe a program designed to address preconception health risks in an outreach setting.

By performing a summative evaluation of this program using the RE-AIM framework, it was determined that this outreach program was feasible to implement. Through this program, many preconception health-related needs were found that can be addressed through pharmacist intervention or counseling. Many barriers preventing program participants from receiving routine healthcare were also identified. Community-based outreach programs can help fill gaps in the provision of preconception care, especially for patients who are not receiving such care in traditional healthcare settings. Programs such as this can also serve to collect on health needs to justify additional interventional programs or studies.

The student pharmacists who volunteered to help with this outreach program gained important experience in patient interviewing and counseling, giving them a chance to strengthen their skills and help “real-world” patients. In addition, this program raised awareness among pharmacists and student pharmacists about the upstream and downstream factors influencing community members’ health. For example, through this program, the pharmacists and student pharmacists gained a greater appreciation for the community members’ challenges in accessing not only healthcare but also healthful foods; by going out into the community, they saw firsthand how some towns did not have full grocery stores that sold fresh produce but instead only had gas stations or convenience stores.

Pharmacists and student pharmacists in the United States have reported being interested in providing preconception services to patients, although many report barriers to providing such care [46,47,48]. While this outreach program was performed under the auspices of a mobile health clinic, such a clinic does not need to be present to implement this program. This type of program can be delivered in places such as community pharmacies and ambulatory primary care practice sites. It can also be performed in a number of other settings such as health departments, parent meetings, women’s organizations, churches, public libraries, college campuses, and health fairs, as well as by groups including healthcare providers, colleges of pharmacy, and professional pharmacy organizations. In addition, supervised student pharmacists can implement this program as part of introductory or advanced pharmacy practice experiences (IPPEs or APPEs) in multiple practice settings; it also can be adapted as an interprofessional education activity.

This pilot project can also be further expanded to offer additional services to participants per pharmacists’ scope of practice. Given particular state and national laws, pharmacists and student pharmacists may be able to administer vaccines recommended in the preconception period, rather than refer the participant to the health department or local community pharmacy. Point-of-care testing and blood pressure screening may be incorporated to help identify health risks unknown to the participant. Pharmacists may also be able to provide chronic disease state management or contraceptive services [5,6,7,18,19]. In addition, participants can be screened for social determinants of health, such as food insecurity, and referred to community resources as appropriate [49]. Collecting feedback from participants, community partners, and individuals staffing the events about the outreach program would also be helpful in identifying any changes needed and ensuring continuous quality improvement [42,43,44].

Future iterations of a similar outreach program may also want to consider the best way to frame and market the event to the public. Qualitative interviews with residents of Hardin County subsequent to this program showed that many viewed “women’s health” as a term exclusive to reproductive health that carried stigma and embarrassment [50]. However, aspects of women’s health addressed through preconception care are comprehensive and focus on all areas of women’s wellness. Therefore, additional research should be done to determine which terms best resonate with rural women to expand the provision of preconception care. Although this outreach program was geared toward women, not all individuals capable of becoming pregnant identify as women [3,5], which should be a consideration for those implementing a similar program. Furthermore, there are opportunities to provide preconception care to men [3,51,52,53] and teenagers [54], so future programs may want to extend participation beyond women aged 18–45 years. Finally, those providing this program in their local communities will want to adapt the program accordingly based on the most prevalent health needs in their population and emerging considerations (e.g., COVID-19 vaccine, marijuana use).

## 5. Conclusions

This paper outlines the structure of an outreach program focused on women’s health and the provision of preconception care by pharmacists and student pharmacists in a rural community. The preconception health needs addressed by the outreach program are ones that pharmacists are equipped to impact, either alone or as members of an interdisciplinary team. This program can be replicated with community or ambulatory care pharmacists as well as colleges or schools of pharmacy to improve women’s health as well as maternal and child health outcomes locally. Outreach programs may help to close gaps in care for those living in medically underserved areas.

## Figures and Tables

**Table 1:** Demographic profile of outreach program participants (n = 141).

Age, Years n (%)	Race/Ethnicity n (%)
18–24: 24 (17.0%)	White, non-Hispanic: 135 (95.8%)
25–34: 63 (44.7%)	Two or more races: 4 (2.8%)
35–45: 54 (38.3%)	White, Hispanic: 2 (1.4%)

**Table 2:** Tier 1 preconception health risks reported by outreach program participants (n = 141).

Characteristic	n (%)
Medical Conditions	
Type 1 or 2 diabetes	3 (2.1%)
Hypertension	13 (9.2%)
Overweight/obesity	112 (79.4%)
Health Behaviors	
Tobacco use	29 (20.6%)
Not taking folic acid or multivitamin daily	94 (66.7%)
Eight or more alcoholic drinks/week	1 (0.7%)
Not exercising at least 30 min for 5 days/week	106 (75.2%)

**Table 3:** Barriers to a physician appointment in the last year reported by outreach program participants (n = 141).

Barrier	n (%)
No health insurance	17 (12.1%)
Unable to get appointment	18 (12.8%)
No transportation	15 (10.6%)
Too many things going on	47 (33.3%)
Could not take time off of work	28 (19.9%)

**Figure A1:**
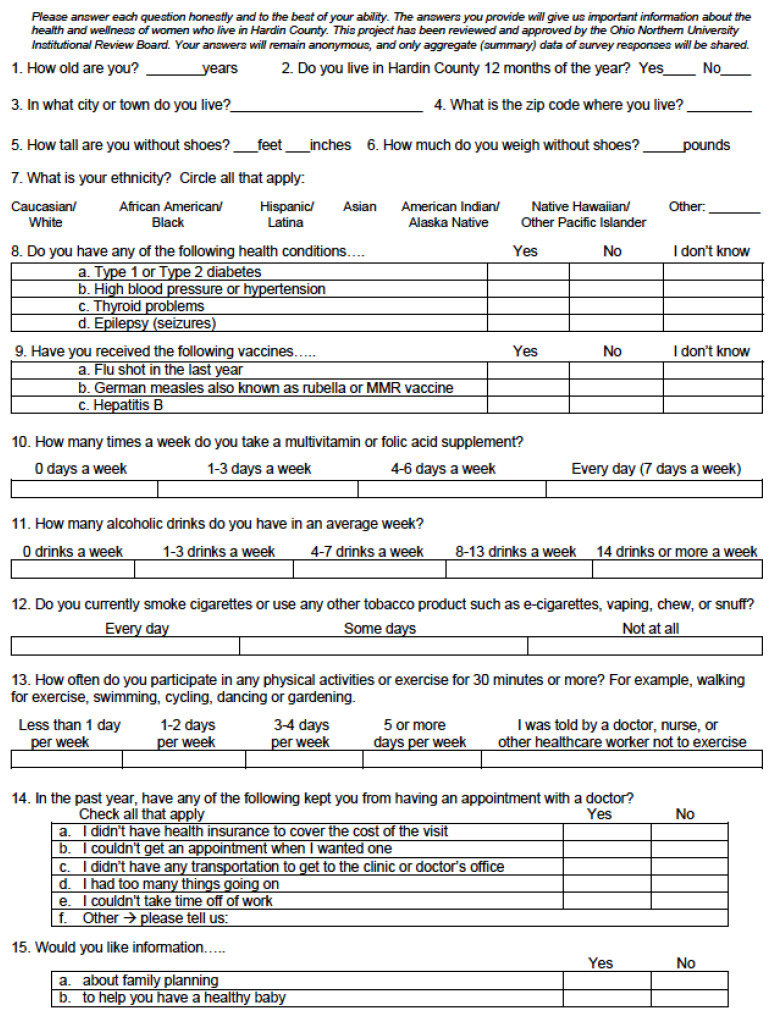
Data Collection Form. Note: Questions on this form were adapted from questionnaires utilized by the Behavioral Risk Factor Surveillance System, Pregnancy Risk Assessment Monitoring System, and Ohio Pregnancy Assessment Survey.

**Figure A2:**
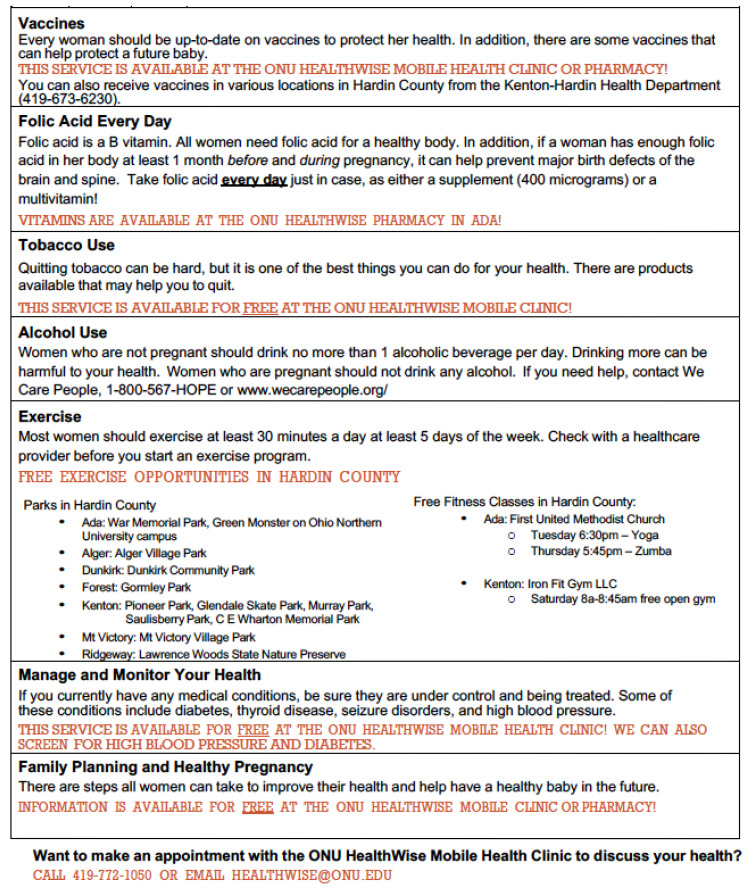
Patient Handout Received during Counseling Session.

**Figure A3:**
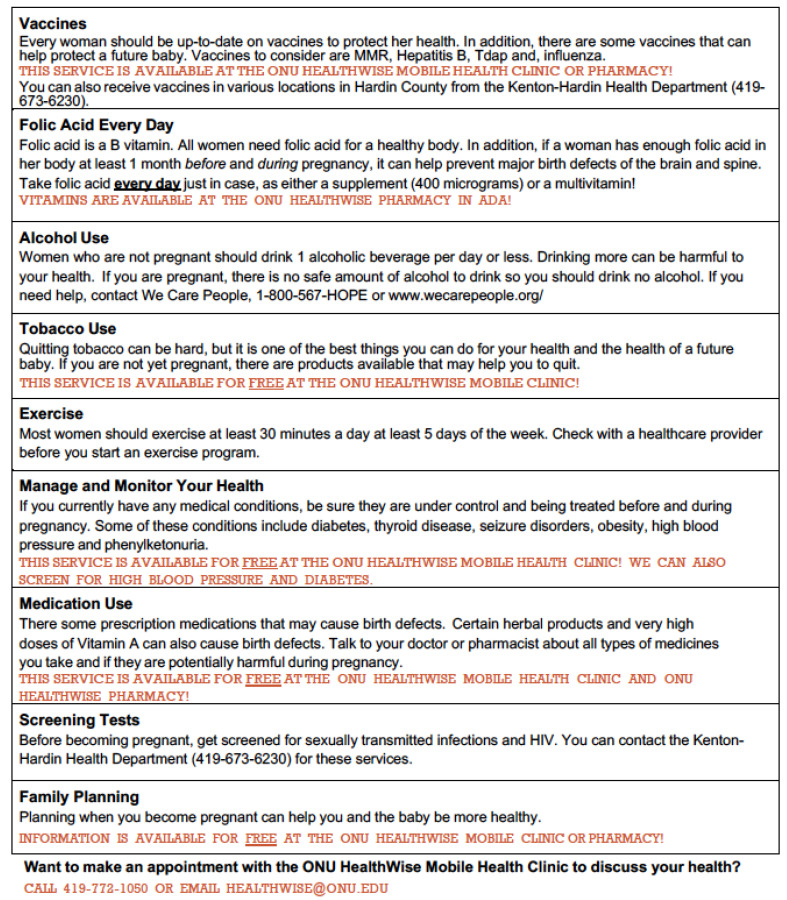
Patient Handout for Patients Wanting More Information about How to Have a Healthy Baby.

## Data Availability

The data presented in this study are available on request from the corresponding author. The data are not publicly available due to its personal nature.
